# Supplementary material for: Developmental Milestones of Infancy and Associations with Later Childhood Neurodevelopmental Outcomes in the Adolescent Brain Cognitive Development (ABCD) Study
Source: Children (Basel). 2022 Sep 20;9(10):1424. doi: 10.3390/children9101424 (PMC9600325; doi:10.3390/children9101424)
Supplement: Supplementary file 1 [file children-09-01424-s001.zip › Supplementary materials-ABCD-CHILDREN-proofread.pdf]

SUPPLEMENTARY MATERIALS

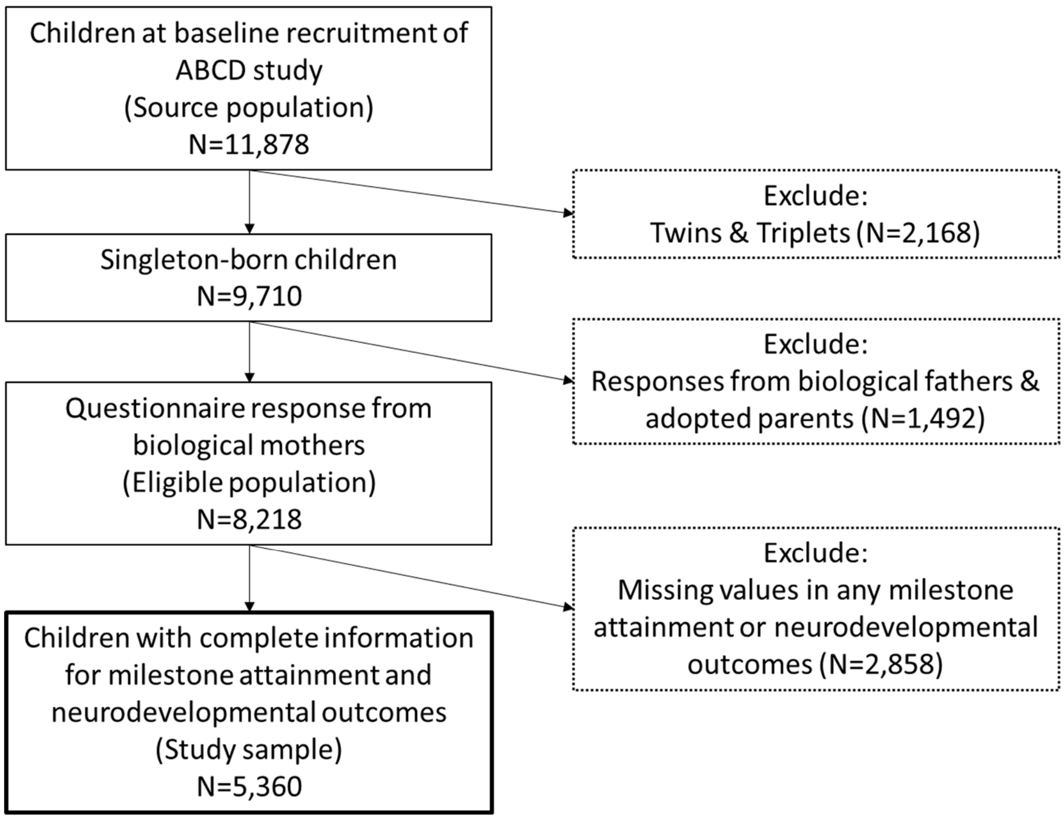

Figure S1. Flow chart of the study sample selection

Table S1. The mean differences in the childhood neurocognitive function scores from the NIH-Toolbox by the delays in infancy milestone attainment.

		Fluid Cognition Composite Score				Crystallized Cognition Composite Score			
	**N=5360**	Adjusted[1] estimate (β1)	95% CI	Adjusted[2] estimate (β2)	95% CI	Adjusted[1] estimate (β1)	95% CI	Adjusted[2] estimate (β2)	95% CI
**Individual milestone attainment**									
Delay of first roll over (in months)									
≥5	802	-1.82	(-2.55, -1.09)	-1.74	(-2.47, -1.01)	-0.80	(-1.26, -0.34)	-0.68	(-1.14, -0.22)
Delay of first sit without assistance (in months)									
≥10	196	-1.16	(-2.54, 0.21)	-1.06	(-2.44, 0.32)	-1.05	(-1.92, -0.19)	-0.94	(-1.81, -0.08)
Delay of speak the first word (in months)									
≥14	378	-1.52	(-2.53, -0.51)	-1.48	(-2.49, -0.47)	-1.28	(-1.92, -0.64)	-1.17	(-1.81, -0.54)
Delay of first walk without assistance (in months)									
≥18	676	-1.35	(-2.13, -0.57)	-1.31	(-2.09, -0.53)	-0.48	(-0.98, 0.01)	-0.47	(-0.96, 0.02)
Multiple milestones attainment									
Delay in motor milestones									
One	1039	-1.13	(-1.79, -0.47)	-1.09	(-1.75, -0.42)	-0.39	(-0.81, 0.03)	-0.31	(-0.73, 0.11)
Two or more	166	-2.52	(-4.00, -1.03)	-2.5	(-3.98, -1.01)	-1.1	(-2.03, -0.16)	-0.98	(-1.91, -0.04)
Delayed in motor milestones and speak the first word									
One + speak	106	-2.37	(-4.21, -0.53)	-2.46	(-4.30, -0.62)	-1.46	(-2.63, -0.30)	-1.4	(-2.57, -0.24)
Two or more + speak	70	-3.3	(-5.55, -1.05)	-3.29	(-5.54, -1.04)	-2.65	(-4.07, -1.23)	-2.44	(-3.86, -1.02)

[1] Adjusted for maternal socio-demographic characteristics (age at delivery, race/ethnicity, marriage status, education level, residential average of family income, self-reported mental health problems) and child's sex at birth

[2] Adjusted for maternal socio-demographic characteristics (age at delivery, race/ethnicity, marriage status, education level, residential average of family income, self-reported mental health problems), child's sex at birth, and perinatal factors (prenatal care utilization, maternal cannabis use/tobacco smoking/alcohol consumption before and after knowing pregnancy, pregnancy-related complications including pre-eclampsia/eclampsia/toxemia, high blood pressure, diabetes, urinary tract infection, severe anemia, and delivery-related outcomes including preterm birth, c-section, jaundice needing treatment, breastfeeding

Table S2. The mean differences in the childhood behavioral problems scores from BPM-Youth by the delays in infancy milestone attainment.

		Internalizing behavioral problems				Externalizing behavioral problems			
	**N=5360**	Adjusted[1] estimate (β1)	95% CI	Adjusted[2] estimate (β2)	95% CI	Adjusted[1] estimate (β1)	95% CI	Adjusted[2] estimate (β2)	95% CI
**Individual milestone attainment**									
Delay of first roll over (in months)									
≥5	802	0.06	(-0.09, 0.22)	0.03	(-0.12, 0.19)	0.03	(-0.11, 0.18)	0.01	(-0.14, 0.15)
Delay of first sit without assistance (in months)									
≥10	196	0.06	(-0.23, 0.35)	0.02	(-0.28, 0.30)	0.23	(-0.05, 0.51)	0.20	(-0.08, 0.48)
Delay of speak the first word (in months)									
≥14	378	0.42	(0.20, 0.63)	0.39	(0.18, 0.61)	0.41	(0.21, 0.62)	0.39	(0.18, 0.59)
Delay of first walk without assistance (in months)									
≥18	676	0.10	(-0.07, 0.26)	0.09	(-0.08, 0.25)	-0.16	(-0.32, 0.00)	-0.17	(-0.33, 0.00)
Multiple milestones attainment									
Delay in motor milestones									
One	1039	0.04	(-0.10, 0.18)	0.03	(-0.11, 0.17)	-0.14	(-0.27, 0.00)	-0.14	(-0.27, 0.00)
Two or more	166	0.14	(-0.17, 0.45)	0.12	(-0.19, 0.43)	0.06	(-0.23, 0.37)	0.03	(-0.27, 0.34)
Delayed in motor milestones and speak the first word									
One + speak	106	0.25	(-0.14, 0.64)	0.24	(-0.15, 0.62)	0.15	(-0.23, 0.53)	0.18	(-0.19, 0.56)
Two or more + speak	70	0.47	(0.01, 0.95)	0.40	(-0.07, 0.88)	0.41	(-0.05, 0.87)	0.33	(-0.13, 0.79)

[1] Adjusted for maternal socio-demographic characteristics (age at delivery, race/ethnicity, marriage status, education level, residential average of family income, self-reported mental health problems) and child's sex at birth

[2] Adjusted for maternal socio-demographic characteristics (age at delivery, race/ethnicity, marriage status, education level, residential average of family income, self-reported mental health problems), child's sex at birth, and perinatal factors (prenatal care utilization, maternal cannabis use/tobacco smoking/alcohol consumption before and after knowing pregnancy, pregnancy-related complications including pre-eclampsia/eclampsia/toxemia, high blood pressure, diabetes, urinary tract infection, severe anemia, and delivery-related outcomes including preterm birth, c-section, jaundice needing treatment, breastfeeding

Table S3. Stratified analyses of infancy developmental milestones and childhood neurocognitive function and behavioral problems by sex

	**Males**			**Females**			**P-interaction**
	**N**	Adjusted estimate (β1)	95% CI	**N**	Adjusted estimate (β1)	95% CI	
Delay of first roll over (≥5 months)							
Fluid cognitive function	447	-2.21	(-3.22, -1.20)	355	-1.28	(-2.33, -0.23)	0.27
Crystallized cognitive function	447	-0.71	(-1.34, -0.07)	355	-0.86	(-1.53, -0.19)	0.68
Internalizing behaviors	447	0.05	(-0.15, 0.26)	355	0.06	(-0.18, 0.29)	0.76
Externalizing behaviors	447	-0.02	(-0.23, 0.19)	355	0.09	(-0.12, 0.30)	0.57
Delay of first sit without assistance (≥10 months)							
Fluid cognitive function	124	-1.60	(-3.39, 0.18)	72	-0.40	(-2.59, 1.78)	0.46
Crystallized cognitive function	124	-1.26	(-2.39, -0.14)	72	-0.72	(-2.11, 0.66)	0.63
Internalizing behaviors	124	0.16	(-0.20, 0.51)	72	-0.06	(-0.55, 0.43)	0.42
Externalizing behaviors	124	0.05	(-0.32, 0.42)	72	0.59	(0.16, 1.02)	0.08
Delay of speak the first word (≥14 months)							
Fluid cognitive function	252	-1.66	(-2.94, -0.38)	126	-1.14	(-2.82, 0.53)	0.64
Crystallized cognitive function	252	-1.23	(-2.03, -0.42)	126	-1.42	(-2.48, -0.37)	0.82
Internalizing behaviors	252	0.42	(0.16, 0.67)	126	0.39	(0.01, 0.76)	0.87
Externalizing behaviors	252	0.35	(0.09, 0.61)	126	0.51	(0.17, 0.84)	0.43
Delay of first walk without assistance (≥18 months)							
Fluid cognitive function	369	-1.56	(-2.65, -0.47)	307	-1.04	(-2.16, 0.07)	0.52
Crystallized cognitive function	369	-0.88	(-1.58, -0.20)	307	0.03	(-0.69, 0.73)	0.08
Internalizing behaviors	369	0.06	(-0.16, 0.28)	307	0.14	(-0.11, 0.39)	0.78
Externalizing behaviors	369	-0.21	(-0.44, 0.02)	307	-0.10	(-0.32, 0.12)	0.52

Adjusted for maternal socio-demographic characteristics (age at delivery, race/ethnicity, marriage status, education level, residential average of family income, self-reported mental health problems) and child's sex at birth

Table S4. The odds ratio (OR) and 95% confidence interval (CI) for latent profiles of childhood neurodevelopment at age 9-10 according to delays in infancy milestones attainment

	N	OR[1]	95%CI
Delay of first roll over (>=5 months)			
Typical behavioral and cognitive outcomes	436	Ref	
Poorer behavioral and cognitive outcomes	113	1.27	(1.01, 1.62)
Poorer behavioral outcomes	93	1.13	(0.89, 1.45)
Poorer cognitive outcomes	120	1.66	(1.28, 2.15)
Better cognitive outcomes	40	1.02	(0.71, 1.45)
Delay of first sit without assistance (>=10 months)			
Typical behavioral and cognitive outcomes	118	Ref	
Poorer behavioral and cognitive outcomes	27	1.19	(0.76, 1.85)
Poorer behavioral outcomes	17	0.74	(0.44, 1.25)
Poorer cognitive outcomes	24	1.19	(0.72, 1.94)
Better cognitive outcomes	10	0.90	(0.46, 1.75)
Delay of speak the first word (>=14 months)			
Typical behavioral and cognitive outcomes	204	Ref	
Poorer behavioral and cognitive outcomes	63	1.95	(1.43, 2.67)
Poorer behavioral outcomes	54	1.49	(1.08, 2.04)
Poorer cognitive outcomes	39	1.47	(0.99, 2.19)
Better cognitive outcomes	18	0.80	(0.48, 1.33)
Delay of first walk without assistance (>=18 months)			
Typical behavioral and cognitive outcomes	392	Ref	
Poorer behavioral and cognitive outcomes	96	1.17	(0.91, 1.51)
Poorer behavioral outcomes	75	1.05	(0.77, 1.31)
Poorer cognitive outcomes	85	1.16	(0.87, 1.54)
Better cognitive outcomes	28	0.70	(0.46, 1.06)

[1] Adjusted for maternal socio-demographic characteristics (age at delivery, race/ethnicity, marriage status, education level, residential average of family income, self-reported mental health problems) and child's sex at birth
